# Diagnostic accuracy of neutrophil-to-lymphocyte ratio in type 2 diabetic nephropathy: a meta-analysis

**DOI:** 10.3389/fendo.2025.1564170

**Published:** 2025-07-22

**Authors:** Yan Wang, Xiaohua Liu, Zhenwen Xiao

**Affiliations:** ^1^ Clinical Laboratory Department, Jinan Third People’s Hospital, Jinan, Shandong, China; ^2^ Department of Nephrology, Jinan Third People’s Hospital, Jinan, Shandong, China

**Keywords:** diabetic nephropathy, early diabetic nephropathy, neutrophils/lymphocytes, meta-analysis, neutrophil-lymphocyte ratio

## Abstract

**Background:**

Diabetic nephropathy (DN) represents one of the most prevalent microvascular complications of type 2 diabetes mellitus (T2DM). The pathogenesis of DN is significantly influenced by the inflammatory response. Thus, the current meta-analysis aimed to assess the diagnostic accuracy of neutrophil-to-lymphocyte ratio (NLR) in early DN and DN.

**Methods:**

Cochrane, Pubmed, Embase, and Web of Science were retrieved from database establishment to August 31, 2024. The Quality Assessment of Diagnostic Accuracy Studies 2 (QUADAS-2) was utilized to assess the quality of included studies. This meta-analysis was carried out via Stata16.0 and Revman 5.3 software.

**Results:**

Finally, this meta-analysis incorporated 18 studies, of which 5 were early DN studies, involving 232 patients with early DN, and 13 were DN studies, involving 4,818 patients with DN. The results indicated that the diagnostic sensitivity of NLR for early DN was 0.83 [95% CI: 0.60-0.94], the specificity was 0.76 [95% CI: 0.61-0.86], and the area under the receiver operating characteristic curve (AUROC) was 0.85 [95% CI: 0.81-0.88]. The diagnostic sensitivity of NLR for DN was 0.73 [95% CI: 0.67-0.79], the specificity was 0.70 [95% CI: 0.59-0.79], and the AUROC was 0.78 [95% CI: 0.74-0.81].

**Conclusions:**

NLR exhibited moderate performance in diagnosing both early DN and DN, and its diagnostic accuracy was higher in early DN than in DN. Due to the limitations of existing studies, further studies are required to verify the findings.

**Systematic Review Registration:**

https://www.crd.york.ac.uk/PROSPERO/, identifier CRD42024591926.

## 1 Background

Globally, type 2 diabetes mellitus (T2DM) is becoming a more severe chronic disease. The International Diabetes Federation (IDF) projects that the global prevalence of diabetes mellitus (DM) will reach 600 million by 2035 (1) and 783 million by 2045 (2). Approximately 40% of T2DM patients will develop diabetic nephropathy (DN), the most severe chronic microvascular complication of the disease (3). Approximately 20%-50% of DM patients have DN, which is the primary cause of end-stage renal disease (ESRD) (4). Currently, the criteria for diagnosing DN include a urinary albumin excretion rate (UAER) ≥ 30 mg/24 h or a random urinary albumin-to-creatinine ratio (UACR) ≥ 30 mg/g and/or an estimated glomerular filtration rate (eGFR) < 60 mL•min-1•(1.73 m^2^)^-1^ (5). However, these indicators have their limitations. First, the calculation of eGFR is mainly based on serum creatinine levels, age, and other intrinsic factors, while serum creatinine levels are strongly influenced by diet and muscle condition, which in turn affect the calculation of eGFR (6). Second, eGFR usually changes when significant kidney damage occurs, and its ability to detect DN in an early stage, when intervention is most beneficial, is limited (7). In addition, there are many factors that influence proteinuria, including hypertension, exercise, high protein diet, fever, urinary tract infection, and congestive heart failure (5). Therefore, new diagnostic indicators are necessary to diagnose DN, given the limitations of eGFR and UACR.

Traditionally, T2DM was considered a chronic metabolic disease, but current study indicates that T2DM is characterized by chronic inflammation (8). Inflammatory indicators such as neuregulin-4 (9), the ratio of serum uric acid to high-density lipoprotein cholesterol (10), and the prognostic nutritional index (11) are associated with microvascular complications and DN in T2DM. The inflammatory response in DN manifests as infiltration of inflammatory cells such as neutrophils, lymphocytes, and macrophages, the release of pro-inflammatory cytokines such as interleukin-1 (IL-1), IL-8, and tumor necrosis factor-a (TNF-a) (12, 13), the production of chemokine (CeC motif) ligand 2 (CCL-2) (14), intercellular adhesion molecule 1 (ICAM-1) (15), transforming growth factor-b (TGF-b), and vascular endothelial growth factor (16), and tissue damage (4). Conversely, the production of these inflammatory factors promotes the infiltration and differentiation of inflammatory cells in kidney tissue (17). Therefore, the role of inflammatory cells in the pathogenesis and diagnosis of DN has become a research hotspot in recent years.

The exploration of more convenient, faster and more economical diagnostic methods has revealed a reliable indicator of systemic inflammation, the neutrophil-to-lymphocyte ratio (NLR), which has a potential to predict cardiovascular diseases and metabolic syndrome (18, 19). Furthermore, NLR is also associated with inflammatory diseases such as thyroid disorders (20), irritable bowel syndrome (21), and T2DM (22). Recent studies indicated the efficacy of NLR in predicting the inflammatory response of DN. A meta-analysis on the correlation of NLR with DN and diabetic retinopathy (DR) demonstrated that compared to T2DM patients and non-DN patients, DN patients had higher NLR values, and NLR was significantly associated with the severity of DN (23). However, current evidence regarding the diagnostic value assessment of NLR is still controversial. The study by Mattared et al. (24) showed that the diagnostic efficacy of NLR for early DN was as high as 0.98. However, the study by Assulyn et al. (25) suggested that the diagnostic efficacy of NLR for early DN was low, only 0.68. The study by Akbas et al. (26) demonstrated that the diagnostic efficacy of NLR for DN was 0.66. Additionally, the available data indicate that NLR exhibited better diagnostic accuracy for low-eGFR, UACR, and DN in T2DM patients in the United States when compared to other hematological indicators (platelets/lymphocytes: PLR, systemic immune inflammation index: SII, monocytes/lymphocytes: MLR, systemic inflammatory response index: SIRI, and total systemic inflammation index: AISI) (27). To sum it up, while the diagnostic value of NLR for DN is unclear in some studies, it has shown a high level of performance in DN diagnosis when compared to other inflammatory markers. Consequently, further studies on the potential role of NLR as a DN-related inflammatory marker are reasonable and well-founded.

Currently, the prediction results of NLR in DN vary greatly, and no study has yet comprehensively analyzed the diagnostic effect of NLR in early DN and DN. Based on the status quo, the current study aimed to collect all available data via an evidence-based meta-analysis and assess the diagnostic accuracy of NLR for early DN and DN, so as to provide recommendations for clinical diagnosis and management of DN.

## 2 Methods

This meta-analysis was conducted in accordance with the Preferred Reporting Items for Systematic Reviews and Meta-analyses Diagnostic Test Accuracy (28). The protocol was registered with the International Prospective Register of Systematic Reviews (PROSPERO, ID: CRD42024591926).

### 2.1 Literature retrieval

Cochrane, Pubmed, Embase, and Web of Science were retrieved up to August 31, 2024. A combination of subject terms and free terms was applied, including: (Diabetic Nephropathies OR Diabetic Kidney Disease OR Diabetic Kidney Diseases OR Diabetic Nephropathy) AND (Neutrophil/Lymphocyte OR Neutrophil/lymphocyte ratio OR Neutrophil-to-lymphocyte ratio OR Neutrophil-lymphocyte ratio). The specific strategy is presented in **Supplementary Table 1**.

### 2.2 Eligibility criteria

Inclusion criteria: 1) Subjects: Adults diagnosed with early DN or DN. 2) Diagnostic indicator: NLR. 3) Golden standard for diagnosis: UAER≥30 mg/24 h or UACR≥30 mg/g, and/or eGFR <60 mL·min^-1^·(1.73 m2)^-1^. When UACR<30 mg/g, the diagnosis is no proteinuria; when albumin-to-creatinine ratio (ACR)=30-300 mg/g, the diagnosis is microproteinuria or early DN; when ACR>300 mg/g, the diagnosis is macroproteinuria or intermediate and advanced DN. 4) Outcome indicators: Obtaining sensitivity, specificity, etc. to evaluate diagnostic accuracy, and considering information from raw data, including true positives, false positives, true negatives, and false negatives. 5) Study type: Observational study, such as case-control or cross-sectional studies.

Exclusion criteria: 1) Reviews, research progress, meeting summaries, pathology reports, correspondence, guidelines, experiences, animal experiments, etc. 2) Articles that are duplicates and cannot be obtained in full text. 3) Articles with inconsistent biomarkers or diseases. 4) Articles with outcome indicators unavailable. 5) Non-English literature.

### 2.3 Literature screening

All retrieved data were entered into Endnote20 software. First, duplicates were removed. Then, according to the eligibility criteria, ineligible studies were removed by reviewing the titles and abstracts. Finally, after reading the full text, studies not meeting the eligibility criteria were removed, and those meeting the criteria were identified. The screening was completed by two investigators (YW. and XHL) independently, and any disagreement was resolved with the participation of a third investigator (ZWX).

### 2.4 Data extraction

Two investigators (YW and XHL) extracted information from the included studies, which comprised basic information on studies (first author, publication year, and country), subjects’ information (patient name, age, gender), and diagnosis method (diagnostic sensitivity and specificity of outcome indicators). Any disagreements during this process were resolved with the third investigator’s (ZWX) participation.

### 2.5 Quality evaluation

Eligible studies were evaluated by two investigators (YW and XHL) for quality and applicability via the QUADAS-2 (Quality Assessment of Diagnostic Accuracy Studies 2) tool (29). The risk of bias (ROB) and clinical applicability were evaluated. The former encompassed experiments to be evaluated, case selection, golden standard, case flow and progression, while the latter incorporated case selection, criteria to be evaluated, and golden standard. The evaluation yielded a low risk for scenarios where the answer of all markers within a range was “yes” and a high risk for scenarios where the answer to any question was “no”. Scenarios lacking sufficient information were designated as “unclear”. Any disagreement arising from the process was addressed with the participation of a third investigator (ZWX). All evaluations were conducted via Revman 5.3 software.

### 2.6 Statistical analysis

The Meta-Disc 1.4 and Stata 16.0 software with the MIDAS module of a bivariate mixed effect model was employed to analyze all diagnosis-related data. The model not only considered factors including threshold effects, sample size, and inter-study heterogeneity, but also kept the bivariate nature of raw data unchanged throughout the process, thereby producing reliable statistical indicators. Forest plots were created to calculate diagnostic odds ratio (DOR), combined sensitivity, specificity, diagnostic score (DS), negative likelihood ratio, and positive likelihood ratio (PLR). Higher DS and DOR values indicated a stronger diagnostic effect. The area under the curve (AUC) was acquired by plotting a summary receiver operating characteristic (SROC) curve. The diagnostic power was categorized as low (AUC=0.5-0.7), medium (AUC=0.7-0.9), and high (AUC=0.9-1.0). Sensitivity analyses were performed to observe the stability of summary statistics and assess how individual studies affected the overall results. To determine the threshold effects, Spearman’s rank correlation coefficient (Spearman ρ) and its corresponding P-value were adopted. *P*>0.05 suggested no heterogeneity among studies due to threshold effects. Heterogeneity was quantified statistically via Higgins *I^2^* and Cochran’s Q test. *P*<0.10 or *I^2^* > 50% indicated high heterogeneity, and a random effect model was adopted. In other cases, a fixed effect model was utilized. When heterogeneity was high, meta-regression and subgroup analysis were performed to determine its sources. Publication bias (PB) was evaluated via a Deeks funnel plot, and *P*<0.05 was considered statistically significant.

## 3 Results

### 3.1 Literature screening

**Figure 1:**
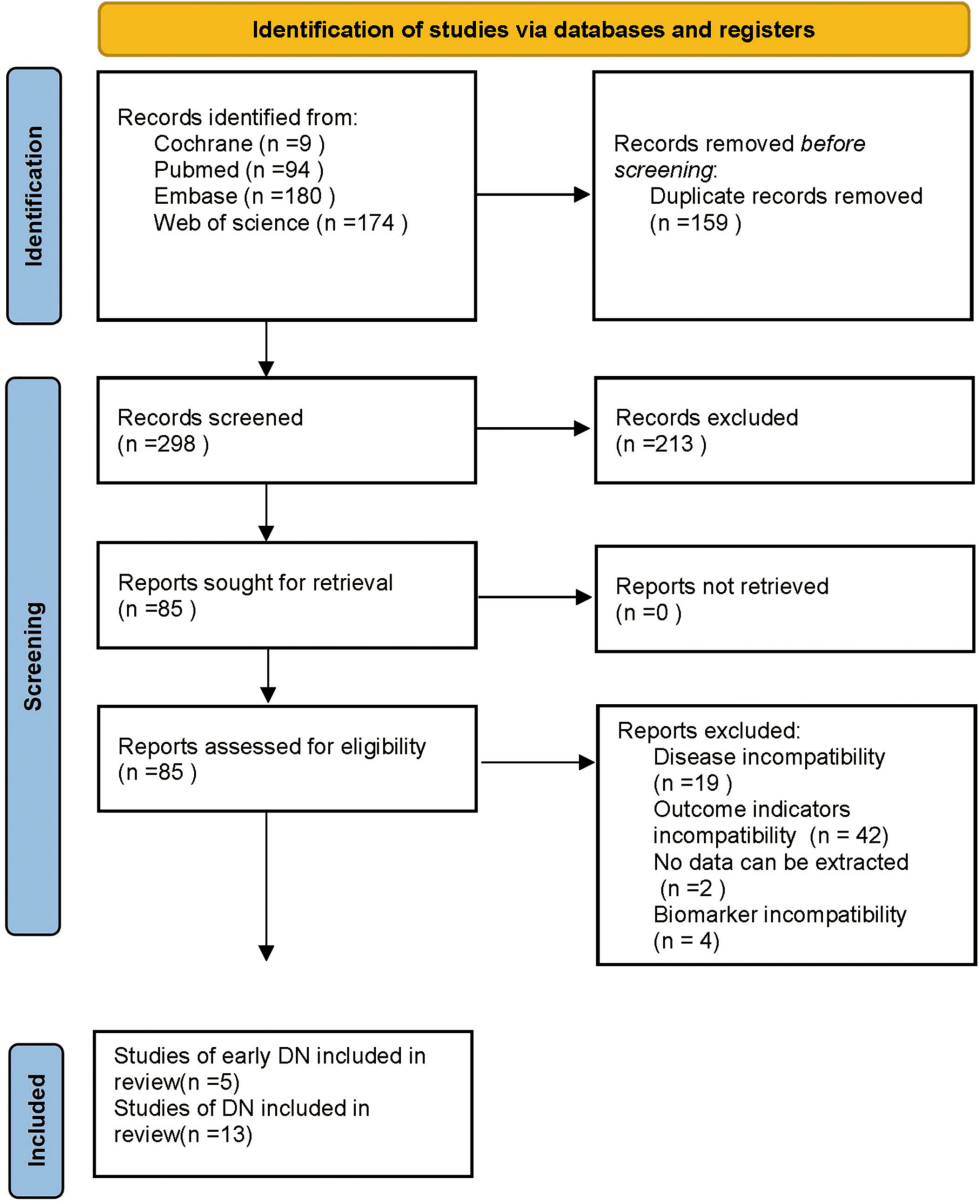
Screening process of articles.

The database retrieval produced 457 articles and after excluding 159 duplicates, 298 remained. Then, 213 articles were removed by reviewing the titles and abstracts in the initial screening, leaving 85 articles. Subsequently, 67 articles were excluded by reviewing the full text, including 19 due to inconsistent disease, 42 due to inconsistent outcome indicators, 2 due to no extractable data, and 4 due to inconsistent biomarkers. Finally, 18 articles were incorporated, including 5 on early DN (24, 25, 30–32) and 13 on DN (26, 27, 33–43). The specifics are presented in **Figure 1**.

### 3.2 Basic characteristics of studies

Eighteen studies (24–27, 30–43) were included in the meta-analysis, with 5 on early DN (24, 25, 30–32) and 13 on DN (26, 27, 33–43). Of the 5 studies on early DN, 4 were conducted in Asia (25, 30–32) and one in Africa (24). The mean age was in a range between 54.8 and 64.0 years, and the NLR cutoff was in a range between 1.56 and 2.54. Of the 13 studies on DN, 12 were conducted in Asia (26, 33–43) a and 1 in North America (27). The mean age was in a range between 48.9 and 71.9 years, and the NLR cutoff was in a range between 1.61 and 3.35. The specifics are outlined in **Table 1**.

**Table 1:** Characteristics of studies.

Study	Country	Study design	Sample size		Age (y)	Gender (F/M)	Disease	NLR cutoff	Diagnostic basis
			Case control						
Chen et al., 2022 (30)	China	CC	49	134	54.8	70/113	Early DN	1.56	UACR
Chollangi et al., 2023 (31)	India	CS	45	45	62.2	37/53	Early DN	2.13	UACR
Mattared et al., 2019 (29)	Egypt	CC	30	50	59.4	_	Early DN	_	24h-UAER
Jaaban et al., 2021 (32)	Syria	CC	50	108	57.1	66/92	Early DN	2.20	UACR
Assulyn et al., 2020 (25)	Israel	CC	58	110	64.0	84/84	Early DN	2.54	UACR, 24h-UAER
Bhattacharyya et al., 2021 (33)	India	CS	39	41	59.9	33/47	DN	2.44	UACR
Akbas et al., 2014 (26)	Turkey	CS	68	132	57.3	103/97	DN	1.70	UACR
Li et al., 2022 (34)	China	CS	365	290	59.9	304/351	DN	2.46	UACR
RAKESH et al., 2024 (35)	India	CS	52	52	53.3	28/76	DN	2.92	UACR
Tutan et al., 2023 (36)	Turkey	CC	108	219	61.0	179/148	DN	1.93	UACR
Fang, et al., 2024 (37)	China	CS	60	30	64.1	43/47	DN	2.24	UACR
Singh et al., 2022 (38)	India	CS	146	390	56.1	_	DN	3.28	24h-UAER
Huang et al., 2017 (39)	China	CS	187	134	56.6	135/186	DN	1.76	UACR, Scr
Ayad M et al., 2017 (43)	Iraq	CS	58	72	59.9	59/71	DN	3.35	UACR
Li et al., 2023 (27)	United States	CS	2271	4882	48.9	3959/3194	DN	1.35	UACR, eGFR
Wan et al., 2020 (40)	China	CS	1222	3575	67.0	2585/2212	DN	1.70	UACR, eGFR
Chittawar et al., 2017 (41)	India	CS	110	155	51.1	144/121	DN	2.00	UACR, eGFR
Gao et al., 2024 (42)	China	CS	132	908	71.9	550/490	DN	1.61	eGFR

CS, Cross-sectional; CC, Case-control; DN, Diabetic nephropathy; UACR, Urinary albumin-to-creatinine ratio; 24 h-UAER, 24 h-urinary albumin excretion rate; Scr, Serum creatinine; eGFR, Estimated glomerular filtration rate.

### 3.3 Quality evaluation

**Figure 2:**
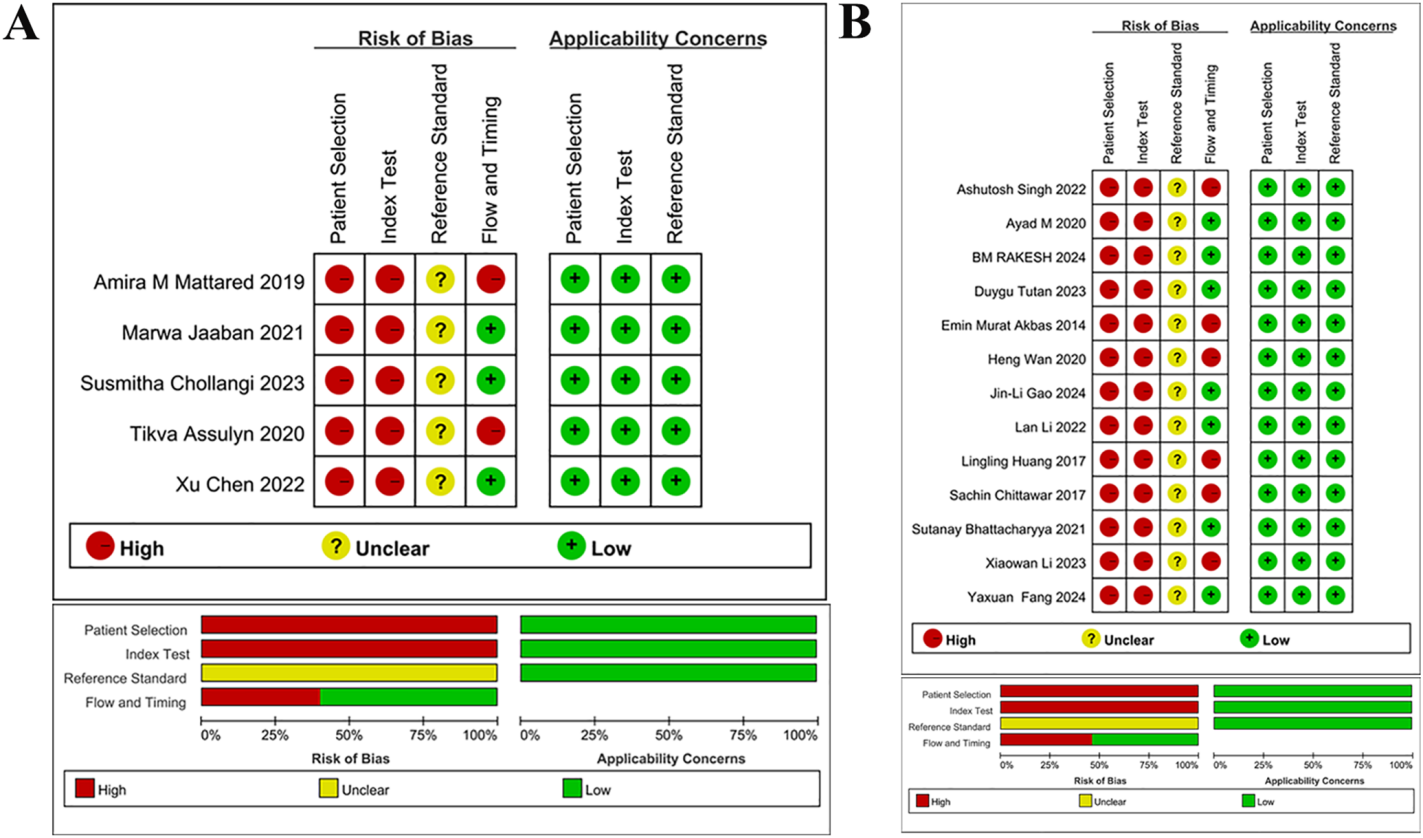
Quality assessment of literature **(A)** Quality assessment for studies on early DN **(B)** Quality assessment for studies on DN Quality assessment of included studies, for each study, ROB and applicability concerns were classified as low, unclear or high. Top: Quality assessment of included studies based on the quality assessment of diagnostic accuracy studies criterion. For each study, ROB and applicability concerns were classified as low, unclear or high. Bottom: Each bar represents the percentage of studies considered as high risk, low risk or unclear for both ROB and applicability concerns.

The quality of studies was assessed via Revman 5.3 software. The results indicated a high risk of bias for all 18 included studies regarding experiments to be evaluated and case selection. For case selection, the reason was an answer of “No” to both “avoiding case-control studies for cases” and “avoiding unreasonable exclusions for studies”. For experiments to be evaluated, the reason was an answer of “No” to “results of experiments to be evaluated were interpreted without knowing the results of golden standard experiments”. All 18 studies yielded an answer of “unclear” to “the golden standard interpretation was blinded”. Two of five studies on early DNs (24, 25) and 6 of 13 studies on DNs (26, 27, 38–41) were at high risk in terms of “case flow and progression”. Notably, all 18 studies were at “low risk” regarding clinical applicability. The quality evaluation is detailed in **Figure 2**.

Quality assessment of included studies, for each study, ROB and applicability concerns were classified as low, unclear or high. Top: Quality assessment of included studies based on the quality assessment of diagnostic accuracy studies criterion. For each study, ROB and applicability concerns were classified as low, unclear or high. Bottom: Each bar represents the percentage of studies considered as high risk, low risk or unclear for both ROB and applicability concerns.

### 3.4 Results of meta-analysis

#### 3.4.1 Diagnostic effect of NLR on early DN

**Figure 3:**
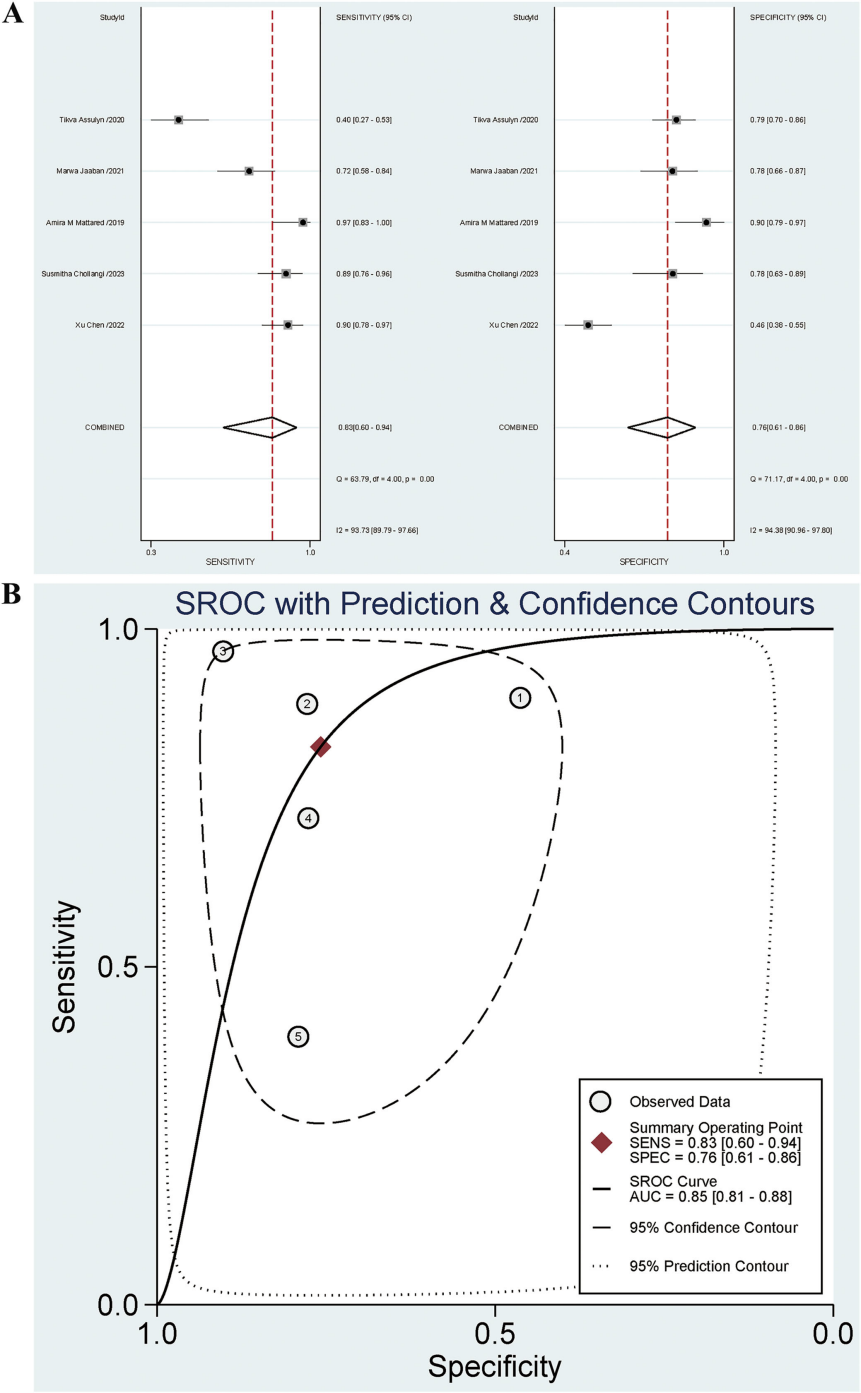
**(A)** Forest plot for diagnostic performance of NLR on early DN; **(B)** AUC for diagnostic performance of NLR on early DN.

Five studies examined the diagnostic effect of NLR in early DN. The sensitivity and specificity of the combined NLR were 0.83 [95% CI: 0.60-0.94] (*I^2^ =* 93.73%) and 0.76 [95% CI: 0.61-0.86] (*I^2^ =* 94.38%), respectively. The PLR, NLR, and DOR were 3.4 [95% CI: 1.9-6.0], 0.23 [95% CI: 0.09-0.60], and 15 [95% CI: 4-56], respectively. The AUC was 0.85 [95% CI: 0.81-0.88]. The forest plot is presented in **Figure 3A**, and the AUC is illustrated in **Figure 3B**. The Spearman ρ was -0.100, and the *P*-value was 0.873, indicating no heterogeneity caused by threshold effects.

Significant heterogeneity was noted. Subgroup analysis was conducted based on blood sugar controlled for negative controls (HbA1c>7% indicating uncontrolled blood sugar), diagnosis of early DN based on UACR, diagnosis of early DN based on a single indicator, and cases from Asia, type of case study to identify potential reasons for heterogeneity. The results indicated that the contributors to a high specificity heterogeneity were cases from Asia (*P*=0.04) and the diagnosis of early DN based on UACR (*P*=0.04). Among these articles, 4 incorporated UACR in the diagnosis, while 1 did not. The specificities were 0.70 [95% CI: 0.58-0.83] and 0.91 [95% CI: 0.78-1.00], respectively. The specificity for articles without UACR in the diagnosis was higher than that for articles with UACR (*P*=0.04), and statistically significant differences were observed. There were 4 articles including cases from Asia and 1 including cases from non-Asia regions. The specificities were 0.91 [95% CI: 0.78-1.00] and 0.70 [95% CI: 0.58-0.83], respectively. The specificity for cases from Asia was higher than that for cases from non-Asia regions (*P*=0.04), and statistically significant differences were observed. The high sensitivity heterogeneity was attributed to the diagnosis of early DN based on a single indicator (*P*=0.01). Four articles included the diagnosis of early DN based on a single indicator and 1 did not. The sensitivities were 0.87 [95% CI: 0.80-0.95] and 0.40 [95% CI: 0.14-0.65], respectively. Furthermore, the sensitivity for articles based on a single indicator outperformed that for articles not based on a single indicator (*P*=0.01), with statistically significant differences observed. The results are illustrated in **Table 2**. The combined sensitivity (Se) for UACR in diagnosing early DN was 0.83 [95% CI: 0.76-0.89], and the specificity (Sp) was 0.61 [95% CI: 0.54-0.67].

**Table 2:** Subgroup analysis for the source of heterogeneity in early DN.

Subgroup factors		No. of articles	Sen.	*P*	Spe.	*P*
Incorporation of UACR in the diagnosis	Yes	4	0.76 [0.58 - 0.95]	0.25	0.70 [0.58-0.83]	**0.04**
	No	1	0.97 [0.90 - 1.00]		0.91 [0.78 - 1.00]	
Single indicator	Yes	4	0.87 [0.80 - 0.95]	**0.01**	0.75 [0.60 - 0.89]	0.62
	No	1	0.40 [0.14 - 0.65]		0.79 [0.55 - 1.00]	
Cases from Asia	Yes	4	0.76 [0.58 - 0.95]	0.25	0.70 [0.58 - 0.83]	**0.04**
	No	1	0.97 [0.90 - 1.00]		0.91 [0.78 - 1.00]	

Bold value indicates that P<0.05 was considered statistically significant.

#### 3.4.2 Diagnostic performance of NLR on DN

**Figure 4:**
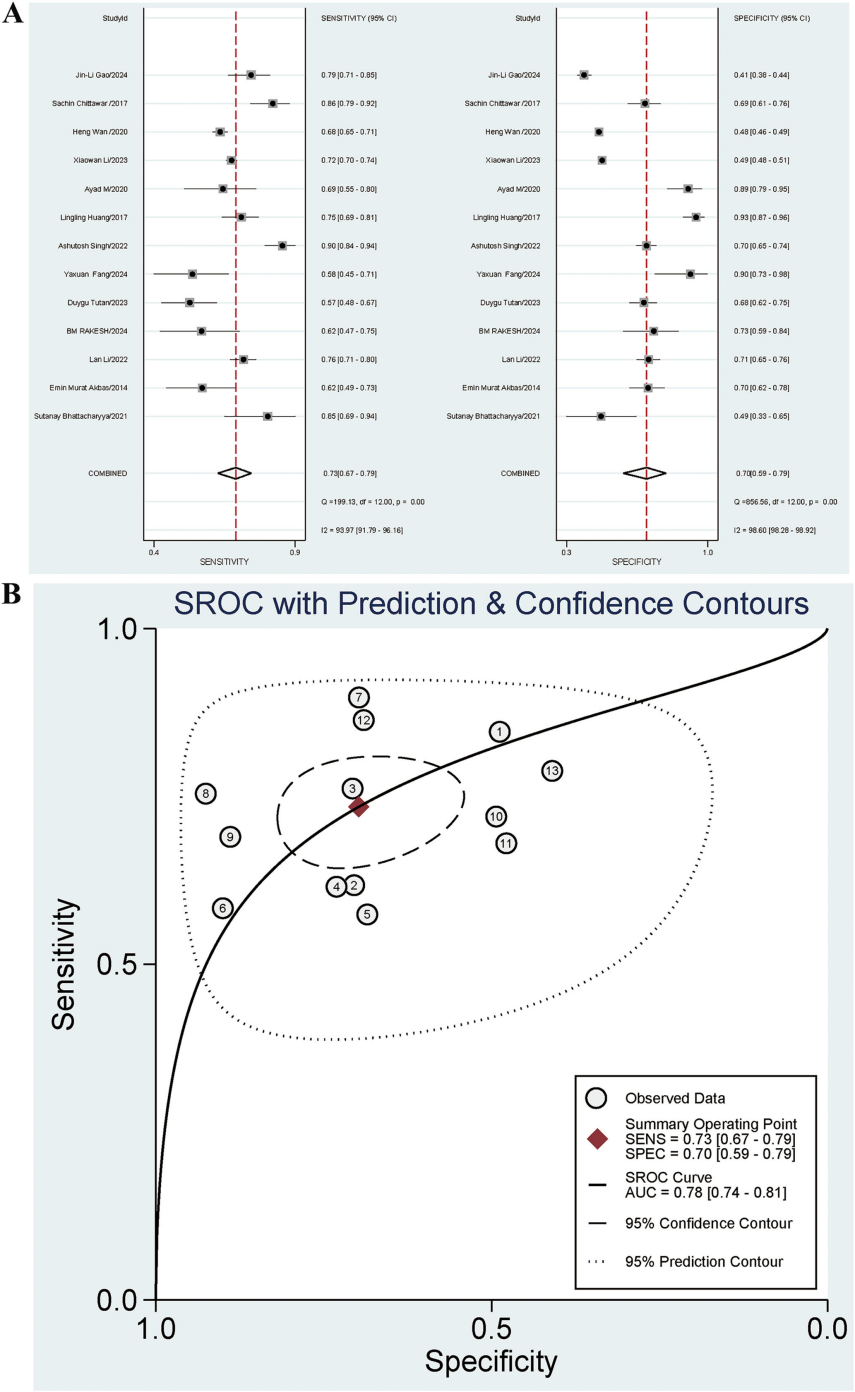
**(A)** Forest plot for diagnostic performance of NLR on DN; **(B)** AUC for diagnostic performance of NLR on DN.

Thirteen studies investigated the diagnostic effect of NLR in DN. The sensitivity and specificity of the combined NLR were 0.73 [95% CI: 0.67-0.79] (*I^2^ =* 93.97%) and 0.70 [95% CI: 0.59-0.79] (*I^2^ =* 98.60%), respectively. The PLR, NLR, and OR were 2.4 [95% CI: 1.8-3.3], 0.38 [95% CI: 0.30-0.48], and 6 [95% CI: 4-11], respectively. The AUC was 0.78 [95% CI: 0.74-0.81]. The forest plot is presented in **Figure 4A**, and the AUC is illustrated in **Figure 4B**. The Spearman ρ was 0.280, and the *P*-value was 0.354, indicating no heterogeneity caused by threshold effects.

The summary indicated significant heterogeneity. Subgroup analysis was performed based on gender, patients from China or Asia, additional complications for negative controls, diagnosis of DN based on UACR, diagnosis of DN based on a single indicator, and type of case study. Regression analysis was conducted based on time, age, country, continent, race, DN diagnosis basis and type of case study to identify possible sources of heterogeneity. The results indicated that patients from China (*P*=0.01) and the diagnosis of DN based on a single indicator (*P*=0.01) were significant contributors to the high sensitivity heterogeneity. Among the articles, 5 involved patients from China and 8 involved patients from non-China regions. The sensitivities were 0.72 [95% CI: 0.63-0.81] and 0.74 [95% CI: 0.67-0.82], respectively. The diagnostic sensitivity for articles including patients from non-China regions was higher than that for articles including patients from China (*P*=0.01), and statistically significant differences were observed. Nine articles included the diagnosis of DN based on a single indicator, and 4 did not. The sensitivities were 0.72 [95% CI: 0.65-0.80] and 0.76 [95% CI: 0.66-0.85], respectively. The sensitivity for articles with the diagnosis of DN not based on a single indicator was higher than that for articles with the diagnosis of DN based on a single indicator (*P*=0.01), and statistically significant differences were observed. The results are presented in **Table 3**. The combined Se for UACR in diagnosing DN was 0.67 [95% CI: 0.60-0.74], and the Sp was 0.74 [95% CI: 0.64-0.82]. The Se for the combined diagnosis of UACR and eGFR was 0.71 [95% CI: 0.70-0.73], and the Sp was 0.49 [95% CI: 0.48-0.50].

**Table 3:** Subgroup and regression analyses for sources of heterogeneity in DN.

Subgroup factors		No. of literature	Sen.	*P*	Spe.	*P*
Patients from China	Yes	5	0.72 [0.63-0.81]	**0.01**	0.72 [0.56-0.87]	0.51
	No	8	0.74 [0.67-0.82]		0.69 [0.56-0.81]	
Single indicator	Yes	9	0.72 [0.65-0.80]	**0.01**	0.71 [0.59-0.82]	0.55
	No	4	0.76 [0.66-0.85]		0.68 [0.50-0.86]	

Bold value indicates that P<0.05 was considered statistically significant.

### 3.5 PB

**Figure 5:**
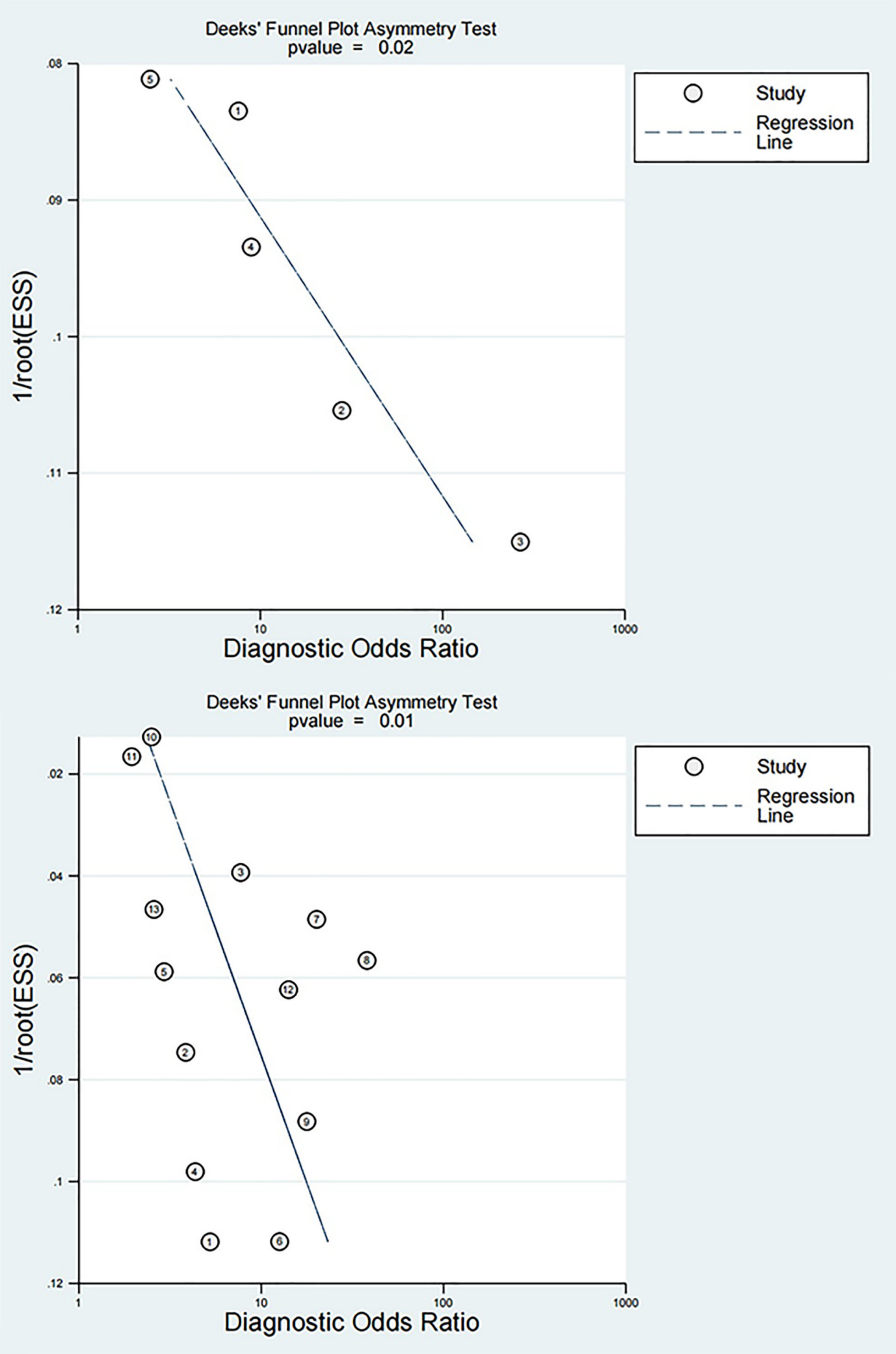
Funnel plots for diagnosis of early DN and DN by NLR The top is the funnel plot for diagnosis of early DN by NLR, and the bottom is the funnel plot for diagnosis of DN by NLR.

PB was visualized via a funnel plot, which indicated PB in the diagnosis of both early DN (*P*=0.02) and DN (*P*=0.01) by NLR, as shown in **Figure 5**.

## 4 Discussion

Recent findings have emphasized the role of glucose dysregulation and systemic inflammation in the development of subclinical organ damage in patients with metabolic disorders. In familial hypercholesterolemia, altered glycemic status has been shown to influence the distribution of atherosclerotic burden, with peripheral vascular injury being more frequent in insulin-resistant individuals (44). Similarly, in the context of acute hyperbilirubinemia, early subclinical renal impairment has been demonstrated using tubular injury biomarkers, even in the absence of overt changes in eGFR or serum creatinine (45). These findings support the rationale for implementing complementary biomarkers to enhance early detection and risk stratification in diabetic nephropathy.

In recent years, studies on the inflammatory response mechanism in DN have led to the development of numerous hematological inflammatory diagnostic indicators for early DN and DN, including red blood cell distribution width (RDW) (31) PLR (27), MLR (27), mean platelet volume (MPV) (46), platelet distribution width (PDW) (46), SII (47), NLR, and others. Among these inflammatory indicators, NLR has received the most research attention. It possesses independent diagnostic value and a higher predictive value for DN (27). The current meta-analysis aimed to determine the diagnostic power of NLR in early DN and DN via an evidence-based approach. A pooled analysis of 18 studies was performed. Of these, 5 studies focused on early DN, involving 232 early DN patients, and 13 studies focused on DN, involving 4,818 DN patients. The results indicated that the diagnostic sensitivity of NLR for early DN was 0.83 [95% CI: 0.60-0.94], the specificity was 0.76 [95% CI: 0.61-0.86], and the AUC was 0.85 [95% CI: 0.81-0.88]. The diagnostic sensitivity of NLR for DN was 0.73 [95% CI: 0.67-0.79], the specificity was 0.70 [95% CI: 0.59-0.79], and the AUC was 0.78 [95% CI: 0.74-0.81]. Since there was high heterogeneity of NLR for both early DN and DN diagnosis, the reasons were explored via subgroup analyses. The results indicated that in the diagnosis of early DN by NLR, the diagnosis of early DN based on UACR (*P*=0.04) and cases from Asia (*P*=0.04) were the sources of high heterogeneity for specificity; the diagnosis of early DN based on a single indicator was the source for high sensitivity heterogeneity (*P*=0.01). In the diagnosis of DN by NLR, patients from China (*P*=0.01) and the diagnosis of DN based on a single indicator (*P*=0.01) were the sources of high heterogeneity for sensitivity.

Metabolic abnormalities, hemodynamic changes, and inflammatory responses are all involved in the pathogenesis of DN, with inflammatory responses playing a significant role (3, 48, 49). Neutrophils (N) are the first line of defense for the innate immune system, responsible for non-specific inflammatory responses that primarily involve phagocytosis and apoptosis (50). First, growth factors, cytokines, and chemokines are elevated in renal biopsies (16). Cytokines, including interleukin-1 (IL-1), IL-6, IL-16, and IL-18, have been implicated in the pathogenesis of DN (3). For example, IL-6 has been shown to recruit N infiltrates in the tubule interstitium, which is linked to podocyte hypertrophy and glomerular basement membrane thickening (51). These changes finally lead to proteinuria and decreased renal function. Secondly, the recruitment of monocytes and macrophages in kidney tissue is a key step in the pathophysiological process of DN (52). Monocytes coordinate the immune cell response at the glomeruli and vascular interface, including the recruitment and activation of N (53). Lymphocytes (L) are primary cells in the adaptive immune response, comprising T lymphocytes and B lymphocytes. Numerous studies have demonstrated the pivotal role of T lymphocytes in the development of DN, and elevated T lymphocyte levels in the blood correlated with UAC (54–56). Lampropoulou et al. (57) found that T lymphocytes and tumor necrosis factor-α (TNF-α) are activated in the early stages of DN. Consequently, N and L have a role in the pathogenesis of DN. NLR refers to the ratio of N count to L count in peripheral blood and is a ratio of chronic inflammation between two different immune pathways. Compared to N count, L count, or white blood cell (WBC) count alone, NLR is less affected by unknown factors of various physiological or pathological states. As a novel, straightforward, and cost-effective inflammatory marker (58), NLR has gained significant attention for its diagnostic accuracy in DN.

Ayad M. Gaidan et al. (43) and Fang et al. (37) indicated the independent predictive value of NLR for DN. Jaaban et al. (32), Gao et al. (42), and Singh et al. (38) demonstrated a positive correlation between NLR and proteinuria levels, as well as a negative correlation between NLR and eGFR levels. Wan et al. (40) revealed that NLR was positively linked to cardiovascular disease and DN in T2DM, while it was not correlated with DR. Chittawar et al. (41) found that NLR performed best in predicting DN, followed by DR. Huang et al. (39) discovered that NLR could be used as a predictor of DN and DR and correlated with the severity of the disease. These findings indicated the clinical value of NLR in the diagnosis of early DN and DN, aligning with this meta-analysis.

DN has two distinct phenotypes. One is the proteinuria phenotype, and renal biopsy studies have indicated that only 30%-50% of patients with T2DM and DN have typical diabetic glomerulopathy. The other is the “atypical diabetic nephropathy pattern”, which is characterized by severe tubulointerstitial and/or arteriole and vascular abnormalities with mild or no glomerulopathy. Notably, not all patients with DN and reduced eGFR experience increased urine protein, and there is no complete consistency between the two (59). The combined use of the two indicators is more sensitive in diagnosing DN than the use of any one alone (5). This finding aligns with the observation in this meta-analysis, i.e., the sensitivity for articles not based on a single indicator was higher than that for articles based on a single indicator in the diagnosis of DN by NLR. However, it was contradictory to the finding in the diagnosis of early DN by NLR. The reasons may be as follows: Firstly, there was only one study not based on a single indicator in the diagnosis of early DN, and the number was limited. Secondly, the diagnostic indicators of early DN in this meta-analysis incorporated UACR or 24 h-UAER, both of which measured the content of urine protein. The indicator eGFR was not adopted, so the diagnosis of early DN cannot play a complementary role. Similarly, in the analysis on the diagnostic accuracy of NLR in early DN, there was only one study on factors such as early DN from Asia and the diagnosis of early DN based on UACR. The limited body of literature may be attributable to the high specificity heterogeneity observed. In the diagnosis of DN by NLR, the high sensitivity heterogeneity was attributable to the factor of patients from China. The reason might be that all Chinese patients were yellow, while individuals from other racial groups were not. Furthermore, the majority of studies in the current meta-analysis focused on Asian populations. Only one study was from Africa, and one was from North America. The high sensitivity heterogeneity may be caused by the differences in race. Thus, it is anticipated that more studies from other continents will be included.

Although NLR, as an independent diagnostic indicator for DN, has certain limitations in practical clinical applications, it can be incorporated into DN diagnostic models along with other indicators to predict DN diagnosis and prognosis. Zhou et al. (60) and Xu et al. (61) developed models that incorporate various indicators, enabling them to make excellent predictions of DN risk factors and diagnosis. Consequently, NLR can be incorporated as a new indicator into these models for adjustment and update to enhance the value of predicting DN. Given the different reference ranges of NLR in different races and countries, as well as variations in laboratory testing techniques, these factors should be fully considered when adding it to predictive modeling studies. However, this provides a new foundation for the development of prediction models.

The primary advantage of this meta-analysis was that it is the first one of its kind to assess the diagnostic power of NLR in early DN and DN via evidence-based methods. However, there are still some limitations. First, given that the included studies were mostly of high risk, primarily involving case-control study types and assessment of results without blinding, this has a certain impact on the interpretation of the results of this study, and the interpretation of the results should be more cautious. The included studies varied significantly in sensitivity and specificity. Despite conducting heterogeneity analyses, no relevant sources were identified based on currently available data. The significant heterogeneity observed may be primarily attributed to the variety of single and combined diagnostic indicators employed for early DN and DN diagnosis. We anticipate the results of future studies on combined diagnostic indicators, which will serve to verify the conclusions of this study. Second, the number of studies on early DN was 5, and more studies are required to gain a better understanding of this condition. Third, the included studies were all in English, which might introduce selection bias. Fourthly, given that NLR is the ratio of neutrophil to lymphocyte counts and ACR the ratio of urinary albumin to creatinine, the reference ranges vary between different countries and races. Additionally, there are differences in the detection values between different instruments, which leads to variability in the detection values of NLR and ACR. Finally, while the impact of NLR cutoffs on results was explored, it was not determined according to current data. The range of NLR cutoffs that have been summarized in this study is from 1.35 to 3.35. Due to the inability to perform subgroup analysis, a specific analysis of several articles with the largest differences in NLR values was conducted. The results indicated that the larger the NLR cutoff, the higher the AUC, sensitivity, and specificity for the diagnosis of early DN and DN. The most effective NLR cutoff was not confirmed, and further exploration in this area is anticipated.

## 5 Conclusions

NLR exhibited moderate diagnostic power for both early DN and DN, with its diagnostic power for early DN being superior to that for DN. It is anticipated that further studies will examine the diagnostic effect of NLR on early DN or DN from aspects of unified cutoffs and disease diagnostic standards.

## Data Availability

The original contributions presented in the study are included in the article/**Supplementary Material**. Further inquiries can be directed to the corresponding author.
