# Supplementary material for: Diagnostic accuracy of neutrophil-to-lymphocyte ratio in type 2 diabetic nephropathy: a meta-analysis
Source: Front Endocrinol (Lausanne). 2025 Jul 22;16:1564170. doi: 10.3389/fendo.2025.1564170 (PMC12321512; doi:10.3389/fendo.2025.1564170)
Supplement: Supplementary file 1 [file Table1.doc]

**Supplementary Material**

**Supplementary File 1** Retrieval strategy

**Pubmed: 94 records**

#1:"Diabetic Nephropathies"[Mesh]

#2: 'diabetes nephropathy'[Title/Abstract] OR 'diabetic kidney disease'[Title/Abstract] OR 'diabetic nephropathies'[Title/Abstract] OR 'diabetic renal disease'[Title/Abstract] OR 'diabetic nephropathy'[Title/Abstract] OR 'Diabetic Kidney Disease'[Title/Abstract] OR 'Diabetic Kidney Diseases'[Title/Abstract] OR 'Diabetic Glomerulosclerosis'[Title/Abstract] OR 'Intracapillary Glomerulosclerosis'[Title/Abstract] OR 'Kimmelstiel Wilson Disease'[Title/Abstract] OR 'Nodular Glomerulosclerosis'[Title/Abstract] OR 'Kimmelstiel Wilson Syndrom'[Title/Abstract]

#3: ("Diabetic Nephropathies"[Mesh]) OR ('diabetes nephropathy'[Title/Abstract] OR 'diabetic kidney disease'[Title/Abstract] OR 'diabetic nephropathies'[Title/Abstract] OR 'diabetic renal disease'[Title/Abstract] OR 'diabetic nephropathy'[Title/Abstract] OR 'Diabetic Kidney Disease'[Title/Abstract] OR 'Diabetic Kidney Diseases'[Title/Abstract] OR 'Diabetic Glomerulosclerosis'[Title/Abstract] OR 'Intracapillary Glomerulosclerosis'[Title/Abstract] OR 'Kimmelstiel Wilson Disease'[Title/Abstract] OR 'Nodular Glomerulosclerosis'[Title/Abstract] OR 'Kimmelstiel Wilson Syndrom'[Title/Abstract])

#4: 'neutrophil to lymphocyte ratio'[Title/Abstract] OR 'neutrophil/lymphocyte ratio'[Title/Abstract] OR 'NLR'[Title/Abstract] OR 'neutrophil lymphocyte ratio'[Title/Abstract] OR 'Neutrophil/Lymphocyte'[Title/Abstract]

#5: (("Diabetic Nephropathies"[Mesh]) OR ('diabetes nephropathy'[Title/Abstract] OR 'diabetic kidney disease'[Title/Abstract] OR 'diabetic nephropathies'[Title/Abstract] OR 'diabetic renal disease'[Title/Abstract] OR 'diabetic nephropathy'[Title/Abstract] OR 'Diabetic Kidney Disease'[Title/Abstract] OR 'Diabetic Kidney Diseases'[Title/Abstract] OR 'Diabetic Glomerulosclerosis'[Title/Abstract] OR 'Intracapillary Glomerulosclerosis'[Title/Abstract] OR 'Kimmelstiel Wilson Disease'[Title/Abstract] OR 'Nodular Glomerulosclerosis'[Title/Abstract] OR 'Kimmelstiel Wilson Syndrom'[Title/Abstract])) AND ('neutrophil to lymphocyte ratio'[Title/Abstract] OR 'neutrophil/lymphocyte ratio'[Title/Abstract] OR 'NLR'[Title/Abstract] OR 'neutrophil lymphocyte ratio'[Title/Abstract] OR 'Neutrophil/Lymphocyte'[Title/Abstract])

**Embase: 180 records**

#1：diabetic nephropathy'/exp

#2：'diabetic kidney diseases':ab,ti OR 'diabetic nephropathy':ab,ti OR 'diabetic glomerulosclerosis':ab,ti OR 'intracapillary glomerulosclerosis':ab,ti OR 'kimmelstiel wilson disease':ab,ti OR 'nodular glomerulosclerosis':ab,ti OR 'kimmelstiel wilson syndrome':ab,ti OR 'diabetes nephropathy':ab,ti OR 'diabetic kidney disease':ab,ti OR 'diabetic nephropathies':ab,ti OR 'diabetic renal disease':ab,ti

#3：neutrophil lymphocyte ratio'/exp

#4: 'neutrophil to lymphocyte ratio':ab,ti OR 'neutrophil/lymphocyte ratio':ab,ti OR 'nlr':ab,ti OR 'neutrophil lymphocyte ratio':ab,ti OR 'neutrophil/lymphocyte':ab,ti

#5：#3 OR #4

#6：#1 OR #2

#7：#5 AND #6

**Cochrane Library: 9 records**

#1：MeSH descriptor: [Diabetic Nephropathies] explode all trees

#2：(Diabetic Kidney Disease):ti,ab,kw OR (Diabetic Kidney Diseases):ti,ab,kw OR (Diabetic Nephropathy):ti,ab,kw OR (Diabetic Glomerulosclerosis):ti,ab,kw OR (Intracapillary Glomerulosclerosis):ti,ab,kw OR (Kimmelstiel Wilson Disease):ti,ab,kw OR (Nodular Glomerulosclerosis):ti,ab,kw OR (Kimmelstiel Wilson Syndrome):ti,ab,kw OR (diabetes nephropathy):ti,ab,kw OR (diabetic kidney disease):ti,ab,kw OR (diabetic nephropathies):ti,ab,kw OR (diabetic renal disease):ti,ab,kw

#3：#1 OR #2

#4：(neutrophil to lymphocyte ratio):ti,ab,kw OR (NLR):ti,ab,kw OR (neutrophil lymphocyte ratio):ti,ab,kw OR (Neutrophil Lymphocyte):ti,ab,kw

#5：#3 AND #4

**WOS: 174 records**

#1: TS=('diabetes nephropathy' OR 'diabetic kidney disease' OR 'diabetic nephropathies' OR 'diabetic renal disease' OR 'diabetic nephropathy' OR 'Diabetic Kidney Disease' OR 'Diabetic Kidney Diseases' OR 'Diabetic Glomerulosclerosis' OR 'Intracapillary Glomerulosclerosis' OR 'Kimmelstiel Wilson Disease' OR 'Nodular Glomerulosclerosis' OR 'Kimmelstiel Wilson Syndrom')

#2: TS=('neutrophil to lymphocyte ratio' OR 'neutrophil/lymphocyte ratio' OR 'NLR' OR 'neutrophil lymphocyte ratio' OR 'Neutrophil/Lymphocyte')

#3: #1 AND #2
